# The Construction and Characterization of Mitochondrial Ferritin Overexpressing Mice

**DOI:** 10.3390/ijms18071518

**Published:** 2017-07-13

**Authors:** Xin Li, Peina Wang, Qiong Wu, Lide Xie, Yanmei Cui, Haiyan Li, Peng Yu, Yan-Zhong Chang

**Affiliations:** 1 Laboratory of Molecular Iron Metabolism, The Key Laboratory of Animal Physiology, Biochemistry and Molecular Biology of Hebei Province, College of Life Science, Hebei Normal University, Shijiazhuang 050024, China; 18333159079@163.com (X.L.); hbsdwpn@163.com (P.W.); 15933623497@163.com (Q.W.); 18330117356@163.com (Y.C.); lihaiyan606@163.com (H.L.); yupeng0311@hebtu.edu.cn (P.Y.); 2 Department of Biomedical Engineering, Chengde Medical University, Chengde 067000, China; xielide65@163.com

**Keywords:** iron, mitochondrial ferritin, overexpression

## Abstract

Mitochondrial ferritin (FtMt) is a H-ferritin-like protein which localizes to mitochondria. Previous studies have shown that this protein can protect mitochondria from iron-induced oxidative damage, while FtMt overexpression in cultured cells decreases cytosolic iron availability and protects against oxidative damage. To investigate the in vivo role of FtMt, we established FtMt overexpressing mice by pro-nucleus microinjection and examined the characteristics of the animals. We first confirmed that the protein levels of FtMt in the transgenic mice were increased compared to wild-type mice. Interestingly, we found no significant differences in the body weights or organ to body weight ratios between wild type and transgenic mice. To determine the effects of FtMt overexpression on baseline murine iron metabolism and hematological indices, we measured serum, heart, liver, spleen, kidney, testis, and brain iron concentrations, liver hepcidin expression and red blood cell parameters. There were no significant differences between wild type and transgenic mice. In conclusion, our results suggest that FtMt overexpressing mice have no significant defects and the overexpression of FtMt does not affect the regulation of iron metabolism significantly in transgenic mice.

## 1. Introduction

Iron is an essential trace element for cell metabolism. Many essential biochemical processes require iron, such as oxygen transport, DNA synthesis, and electron transport [1,2]. In the brain, iron homeostasis is tightly regulated. Dysregulation of brain iron homeostasis can lead to severe pathological changes in the nervous system [3,4]. Iron deficiency can (1) affect neurotransmitter synthesis and cause language and motion deficiencies during brain development, (2) trigger iron deficiency anemia [5], and (3) be the underlying disorder in other diseases [6,7]. In addition, iron overload can lead to the death of neurons and induce neurodegenerative diseases such as Parkinson’s disease (PD) [8] and Alzheimer disease (AD) [9]. Excess iron, left unchecked, can catalyze the production of hydrogen peroxide (H_2_O_2_) and other damaging reactive oxygen species (ROS), including the highly reactive hydroxyl radical, through a Fenton reaction [10]. The formation of an unmanageable level of hydroxyl radicals generated as the result of ferrous iron accumulation can lead to a cascade of reactions, culminating in the destruction of cell structure, and leading to cell damage. 

Mitochondrial ferritin (FtMt) is a mitochondria-localized iron storage protein encoded by an intron-less gene on chromosome 5q23.1 [11]. Levi et al. [11] have found that FtMt possesses high homology to H-ferritin with ferroxidase activity [12,13], which can help to store iron in the shell structure of FtMt. In contrast to H-ferritin, FtMt lacks an iron responsive element (IRE) consensus sequence for iron-dependent translational control. Thus, the translation of mitochondrial ferritin is not regulated by the IRE–IRP machinery, which generally controls cellular iron homeostasis [11,14,15]. FtMt is mainly expressed in cells with high oxygen consumption and high metabolic activity, such as spermatocytes, neurons, and cardiomyocytes [16]. Additionally, the mitochondrial demand for iron shows a significantly increasing trend as cellular respiration increases in cells. However, there is relatively low expression of FtMt in the liver and spleen, which store considerable amounts of iron in the form of cytosolic ferritin [14]. Studies in HeLa and H1299 cells overexpressing FtMt have shown that the potency of FtMt as a sink for iron is greater than that of cytoplasmic ferritin and that the sequestration of iron in overexpressed FtMt elicits a cellular iron-deficient phenotype [14,17,18].

FtMt may exert a protective role in mitochondria against iron-dependent oxidative damage. It has been found that in some neurodegenerative diseases, such as AD, PD, and Friedreich’s ataxia, the expression of FtMt is induced [19,20]. Furthermore, our previous studies have shown that FtMt can decrease the cellular damage induced by 6-hydroxydopamine- (6-OHDA-) [21], and attenuate Aβ-induced neurotoxicity in PD pathogenesis [22,23].

At present, the specific mechanisms regulating FtMt in iron metabolism are not clear. To investigate the in vivo role of FtMt, we established and characterized FtMt-overexpressing mice. We examined baseline murine iron metabolism and hematological phenotype. Surprisingly, our data show no overt phenotypic differences between wild type and transgenic mice.

## 2. Results

### 2.1. Generation of Transgenic Mice 

**Figure 1:**
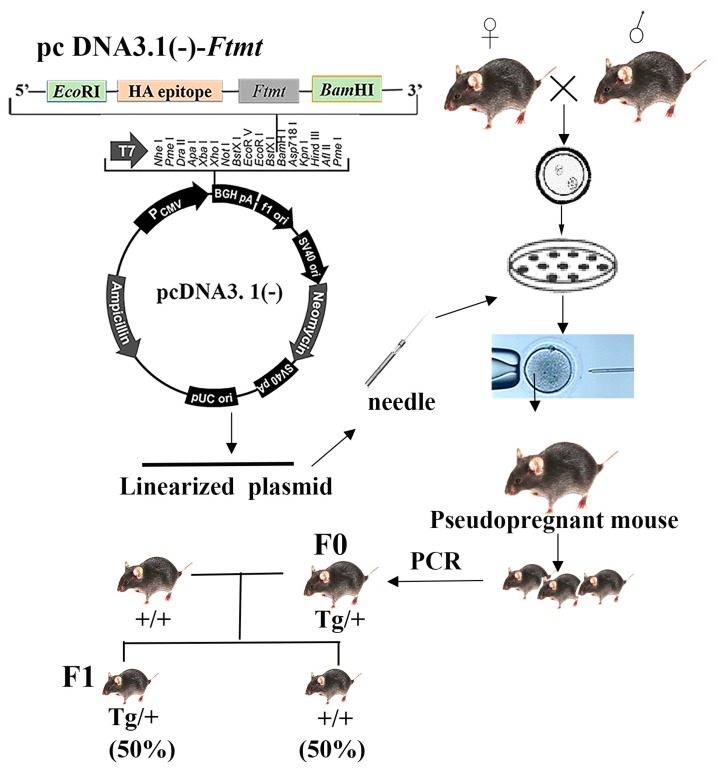
The construction of the transgenic mice. Tg/+, heterozygous; +/+, wild type.

C57BL/6 FtMt overexpressing mice were established by pro-nucleus microinjection with the PiggyBac (PB) System by Cyagen Biosciences Inc. (Suzhou, China). A mouse *Ftmt*-pcDNA3.1(−) construct was established as previously described [21]. Briefly, mouse *Ftmt* cDNA encoding the full *Ftmt* open reading frame was amplified by polymerase chain reaction using 5′-AGGGAATTCACCATGGGCCTGTCCTGCTTTTGGTTCTTCTC-3′, and 5′-GGCGGATCCTATTTAAGCGTAATCTGGAACATCGTATGGGTAGTGCTTGCTCTCGCTTCCAA-3′ primers. The PCR product was inserted into the pGEM-T vector to obtain the plasmid containing a 768-bp fragment including the entire *Ftmt* sequence with a C-terminal hemagglutinin (HA) epitope and an *Eco*RI site at the 5′ end, and a *Bam*HI site downstream of the stop codon at the 3′ end. The 768-bp fragment was excised and sub-cloned into the *Eco*RI/*Bam*HI sites of pcDNA3.1(−) to obtain pcDNA3.1(−)-*Ftmt* [18] (Figure 1). The linearized and purified plasmid was diluted to 1 to 5 ng/μL before it was loaded into the microinjection needle. Morphologically normal zygotes were selected to perform the microinjection and then cultured in an incubator for 1 h. Next, the embryos were transplanted into pseudo-pregnant female mice where the development of embryo proceeded to term, twenty days following the procedure [24]. The pups were genotyped by PCR and positive male mice were viable (F_0_). The foreign gene is usually incorporated into only one chromosome, so the founder mouse is heterozygous. Heterozygous offspring (F_1_) of both sexes were obtained through founder male mouse crosses with wild-type female mice (Figure 1). We were able to obtain the homozygous mice by sib mating of the heterozygous ones. Litters were genotyped to identify male Tg mice to be used in experiments. 

### 2.2. Expression of FtMt is Increased in the Transgenic Mice

**Figure 2:**
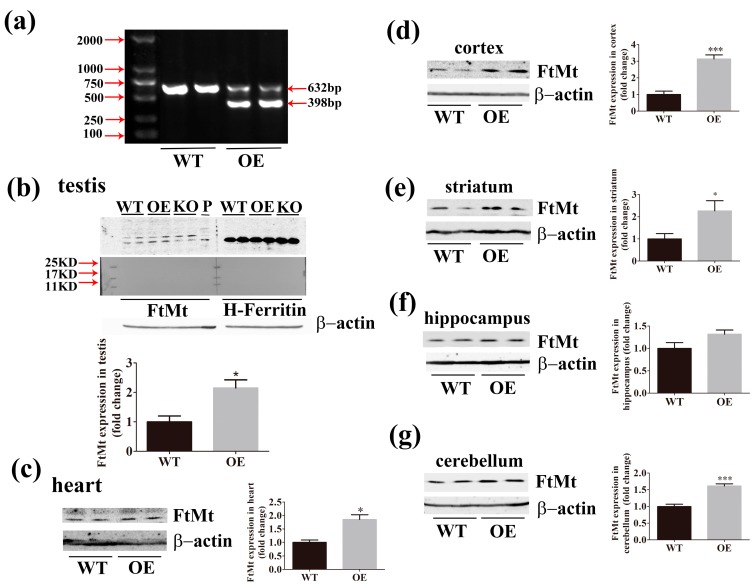
The expression of Mitochondrial ferritin (FtMt) in transgenic mice. (**a**) Genotyping agarose gel electrophoresis analysis of wild-type (WT) and overexpression (OE) mice. Transgenic mice were identified by the presence of 398 and 632 bp PCR products. (**b**–**g**) Western blot and subsequent densitometric analysis of FtMt in (**b**) testis, (**c**) heart, (**d**) cortex, (**e**) striatum, (**f**) hippocampus, (**g**) cerebellum. *n* = 3, * *p* < 0.05, *** *p* < 0.001 versus WT group.

After PCR genotyping FtMt overexpressing mice (Figure 2a), we used western blot analysis to measure FtMt expression. As FtMt has high homology with H-Ferritin, the specificity of the FtMt antibody was confirmed by the detection of FtMt and H-Ferritin from testis with anti-FtMt and anti-H-Ferritin antibodies, respectively [25] (Figure 2b). Using the anti-FtMt antibody to decorate western blots, we observed an increased expression of FtMt in transgenic mice compared to wild-type mice in different regions of brain including cerebellum, cortex, and striatum. Although there was no significant difference in the hippocampus, the expression of FtMt showed a rising trend in this region. The expression of FtMt in the testis and heart also increased in the transgenic mice (Figure 2b–g). Protein extracted from FtMt-overexpressing SH-SY5Y cells was used as positive controls, while extracts from FtMt-deficient mice served as negative controls. Taken together, our results demonstrate the successful generation of FtMt-overexpressing mice. 

### 2.3. FtMt Overexpressing Mice Have Normal Body and Organ Weights 

**Figure 3:**
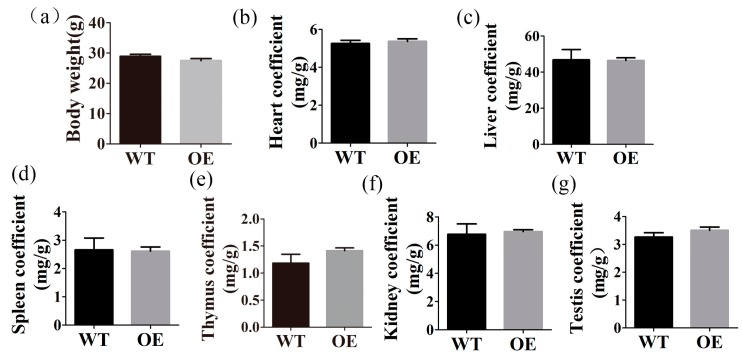
Body weights and organ to body weight ratios in transgenic mice. (**a**) The weights of age-matched (5-month-old) FtMt overexpressing (*n* = 17) and wild-type (*n* = 16) mice were examined. (**b**–**g**) The ratios of heart, liver, spleen, thymus, kidney, and testis weight to body weight ratios were determined in FtMt overexpressing (*n* = 11) and wild-type (*n* = 10) mice. The data are presented as the mean ± SEM.

We determined the body weights of sixteen wild type and seventeen transgenic mice and found that there were no significant differences between the two groups (Figure 3a). We also analyzed the organ to body weight ratios of the mice. The results showed that there were no significant differences in heart, liver, spleen, thymus, kidney, and testis (Figure 3b–g). 

### 2.4. FtMt Overexpressing Mice Exhibit Normal Hematological Parameters and Blood Pressure

**Figure 4:**
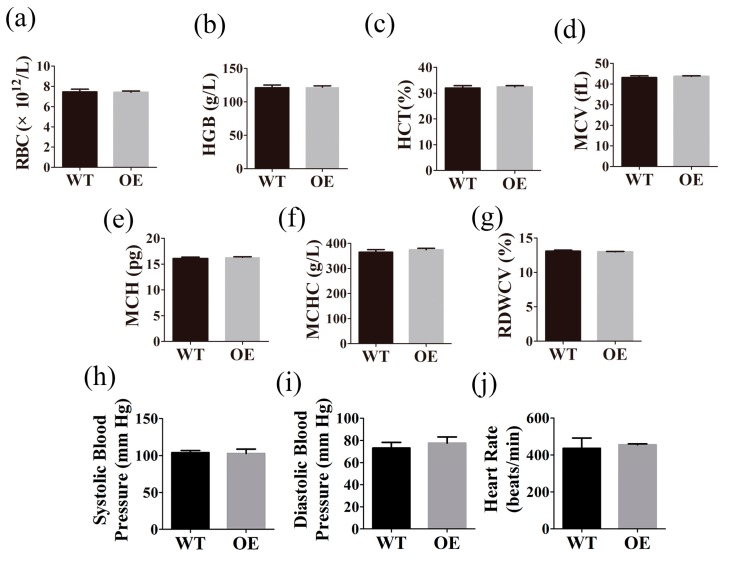
Hematological parameters and blood pressure in the wild type (WT) and transgenic mice (OE). 50 μL fresh blood was obtained from each mouse for hematological parameters measurement. Parameters measured were (**a**) Red blood cell (RBC) count, (**b**) Hemoglobin (HGB), (**c**) Hematocrit (HCT), (**d**) Mean corpuscular volume (MCV), (**e**) Mean corpuscular hemoglobin (MCH), (**f**) Mean corpuscular hemoglobin concentration (MCHC), and (**g**) Coefficient variation of red blood cell volume distribution width (RDWCV). Wild type mice, *n* = 16; transgenic mice, *n* = 17. Data of blood pressure in WT and OE mice: (**h**) Systolic blood pressure, (**i**) Diastolic blood pressure, (**j**) Heart rate, *n* = 3. The data are presented as the mean ± SEM.

To determine the effect of FtMt overexpression on murine hematology, we examined the red blood cell parameters in wild-type and transgenic mice. The parameters we analyzed were the following: red blood cell count (RBC, ×10^12^/L), hemoglobin (HGB, g/L), hematocrit (HCT, %), mean corpuscular hemoglobin (MCH, pg), mean corpuscular hemoglobin concentration (MCHC, g/L), mean corpuscular volume (MCV, fL) and coefficient variation of red blood cell volume distribution width (RDW-CV, %). We found no significant differences (Figure 4). We also measured the blood pressures of the two groups of mice. No significant differences were found in systolic or diastolic blood pressures and heart rates between the two groups. 

### 2.5. Transgenic Mice Have Normal Iron Distribution

**Figure 5:**
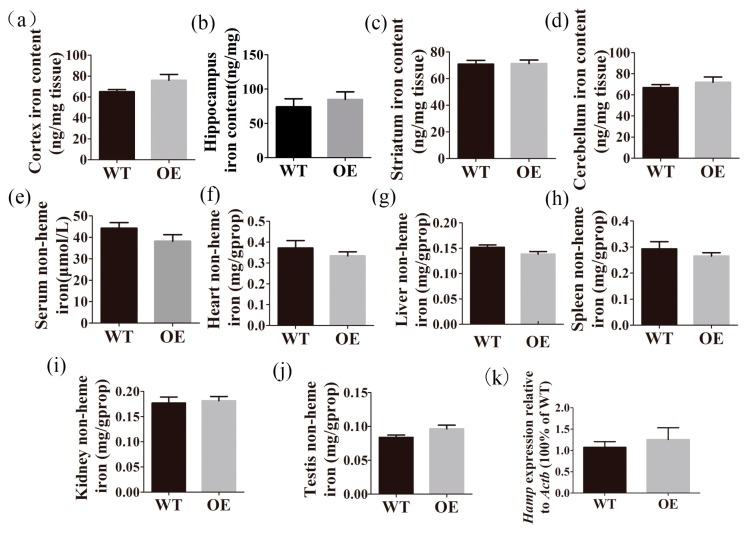
Iron distribution in transgenic mice. The total iron content in the (**a**) cortex, (**b**) hippocampus, (**c**) striatum, (**d**) cerebellum was measured by inductively coupled plasma mass spectrometry (ICP-MS). The total iron concentration in (**e**) serum, (**f**) heart, (**g**) liver, (**h**) spleen, (**i**) kidney, and (**j**) testis. (**k**) *Hamp* expression in liver. Six wild-type and six FtMt overexpression mice were analyzed. The data are presented as the mean ± SEM.

To determine the effect of FtMt overexpression on baseline murine iron metabolism, we measured the iron concentrations in the serum, heart, liver, spleen, kidney, testis, cortex, hippocampus, striatum and cerebellum (Figure 5). We found no significant changes between wild-type and FtMt overexpressing mice. Additionally, the expression of hepcidin in the liver was unchanged between the two groups.

### 2.6. The Effects of FtMt Overexpression on the Levels of Ferritin, TfR1, FPN1, and DMT1

**Figure 6:**
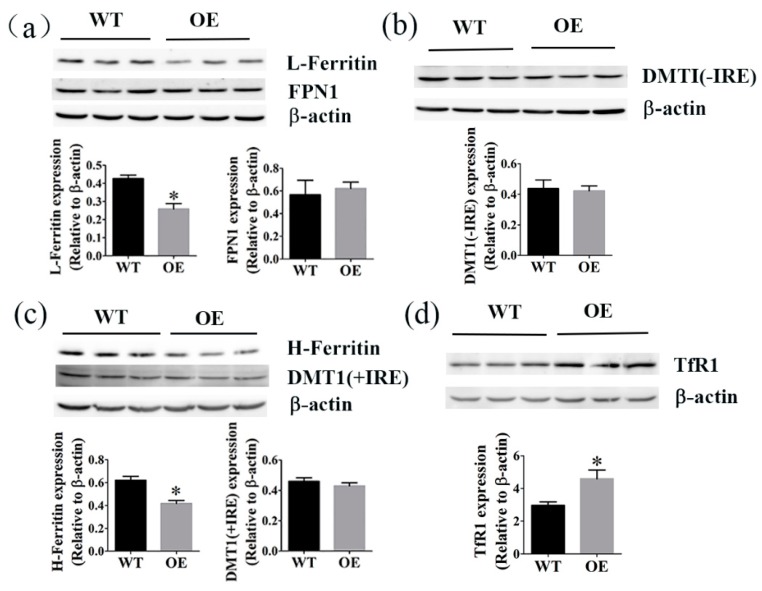
The effect of FtMt overexpression on Ferritin, TfR1, FPN1 and DMT1. Western blot analysis, and subsequent densitometry of (**a**) L-Ferritin, FPN1, (**b**) DMT1(−IRE), (**c**) H-Ferritin, DMT1(+IRE), (**d**) TfR1 in testis. The data are presented as the mean ± SEM, *n* = 3. * *p* < 0.05 versus WT group.

We next examined the levels of Ferritin, TfR1, FPN1, and DMT1 in testis of wild type and transgenic mice. The levels of L-Ferritin and H-Ferritin in FtMt overexpressing mice were decreased significantly (Figure 6a,c), and those of TfR1 increased (Figure 6d), compared to wild-type mice. There were no significant differences in the expression of FPN1, DMTI(+IRE), and DMTI(−IRE) between the two groups (Figure 6a–c). 

### 2.7. The Locomotor Activity Was Not Changed in FtMt Overexpressing Mice 

**Figure 7:**
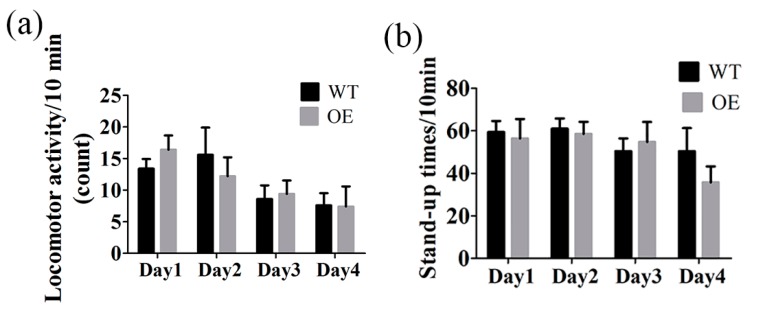
Locomotor activity of wild type (WT) and transgenic mice (OE). (**a**) Locomotor activity counts in ten minutes, (**b**) Stand-up times in ten minutes. The data are presented as the mean ± SEM, *n* = 5.

Our data showed that FtMt overexpressing mice exhibited normal body weights, blood pressure and hematological parameters. The iron contents of brain, liver, testis, heart, kidney and spleen were also not significantly changed in transgenic mice. Was there any behavioral differences between the two types of mice? We tested the activities of the two groups of mice using a mouse autonomic activity tester. The locomotor activity counts and stand-up times of the mice in ten minutes were measured. The data showed that there were no significant differences between wild type and transgenic mice. (Figure 7).

## 3. Discussion

As an essential trace element in biology, iron participates in numerous metabolic processes in humans. The metal plays an important role in oxygen transport, redox reactions, neurotransmitter synthesis, and many other fundamental metabolic processes in cells and organisms [26,27,28]. Though it is essential to mitochondrial and cellular function, iron can also be toxic when in excess through the Fenton reaction, which produces ROS. The labile plasma iron catalyzing this reaction is able to target cell membranes, a process that is considered the main culprit for iron-related cellular and organ damage [29,30,31]. Furthermore, excess iron is considered a contributing neurotoxic factor in several neurodegenerative disorders, including AD [32]. Thus, the levels of iron must be tightly controlled to prevent cellular damage in the brain and maintain normal functions throughout the body.

Mitochondria is an essential and dynamic component of cellular biochemistry. Numerous studies have shown these organelles to be vital to iron metabolism, not only for heme synthesis but also for the biogenesis of [Fe-S] clusters [33]. FtMt, an H-ferritin-like protein targeted to mitochondria, has been shown to protect mitochondria from iron-induced oxidative damage in cells with high metabolic activity and oxygen consumption [23]. Overexpression of FtMt can modulate intracellular iron distribution to decrease cytosolic iron and protect cultured cells against oxidative damage [17,18,21]. Thus, a better understanding of the role of FtMt in mitochondrial iron homeostasis may provide new insights into the treatment of diseases associated with abnormal iron homeostasis.

In order to investigate the in vivo role of FtMt, we established a FtMt overexpressing mouse by pro-nucleus microinjection of pcDNA3.1(−)-*Ftmt* and characterized the animal model. Previous studies have shown that FtMt is highly expressed in the testis in mammals. It is also expressed in the kidney, heart, brain and thymus in mice [34]. There is a relationship between the high metabolic rate of tissues and FtMt expression [14]. Therefore, we determined the expression of FtMt in the heart, testis and four areas of the brain including the cerebellum, cortex, striatum and hippocampus. We found that the FtMt was significantly overexpressed in the heart, testis and the areas of the cortex, striatum, and cerebellum of brain. Interestingly, the increased level of FtMt in different regions of the brain is different, and there is especially no significant change in the hippocampus. It is probably due to the fact that the cell types from different regions are various, which induces different degrees of effect on FtMt overexpression. There may have been some mechanisms involved in the regulation of FtMt overexpression in disparate regions and tissue. Further study needs to be explored. In our study, FtMt overexpression did not affect the hematological indices, blood pressure and heart rates of the mice. There was an increased tendency of iron content in the testis, and a decreased tendency in serum iron, though there were no significant differences between these under baseline conditions. We tested the locomotor activities of the two groups, and we found no behavioral differences in our transgenic mice. In general, FtMt overexpressing mice are healthy and do not show any overt phenotypic differences from wild types under baseline feeding conditions at the age of five months.

We next considered whether overexpression of FtMt altered the iron metabolism in the transgenic mice by measuring the levels of Ferritin, TfR1, FPN1, and DMT1 in the testis, where FtMt is normally highly expressed. Ferritin is the major intracellular iron storage protein [35,36]. It plays an important role in iron metabolism, sequestering excess cytosolic iron and serving as a systemic iron storage depot. Ferritin is composed of two subunits, H and L chains. In ferritin shells, the H subunit mainly exerts an important protective function against oxidative damage because of the ferroxidase activity that converts soluble, catalytic ferrous ions into inert ferric hydroxides [37,38,39]. The L chain lacks the ferroxidase center, but it provides sites for iron nucleation and mineralization, facilitating efficient iron storage [18,40,41]. The Fe-binding protein, Transferrin (Tf) is the major vehicle for iron transport in the body with the help of transferrin receptor 1 (TfR1) [28,42]. And the iron enters cells in the form of receptor-mediated endocytosis of the Tf-TfR1-Fe complex. A reduction in endosomal PH mediates the release of Fe from Tf.

Here we showed that FtMt overexpressing mice, as compared to the wild type, had decreased L-Ferritin and H-Ferritin expression, and increased TfR1 expression. This phenotype was consistent with a deficiency of iron in the cytoplasm, which stimulated a decrease in the levels of L- and H-Ferritin translation in HeLa cells overexpressing human FtMt [17,18]. We suspected that the overexpression of FtMt might cause a redistribution of cellular iron from the cytoplasm to the mitochondria. The iron from the blood was then transported to the cytoplasm with the help of Tf-TfR1 to maintain the balance of iron in the cytoplasm. And there was an increased tendency of iron content in testis. There were no significant differences in the expression of FPN1, DMT1(+IRE) and DMT1(−IRE). In total, the overexpression of FtMt did not affect the regulation of iron metabolism significantly in the transgenic mice.

Our results showed that there were no significant differences in body weight or the ratio of organs to body weight in FtMt overexpressing mice. The changes to the hematological parameters, blood pressure and iron contents were not noticeable. Thus, in normal conditions, the overexpression of FtMt in transgenic mice has no obvious effects compared to wild type mice. Bartnikas et al. [43] found that there were no significant defects in mice lacking mitochondrial ferritin. Our previous studies [44] showed that FtMt disruption decreased the exhaustion exercise time and altered heart morphology with severe cardiac mitochondrial injury. The absence of FtMt increased the sensitivity of mitochondria to cardiac injury via oxidative stress. It suggests that FtMt has a protective role regarding cell damage when responding to oxidative stress. Therefore, we speculate that FtMt overexpression may play a very important role when one suffers from the risk.

In conclusion, there were no pronounced differences between the wild type and FtMt overexpressing mice. The transgenic mice did not exhibit abnormalities, and iron metabolism was also not significantly influenced Generating FtMt overexpressing mice can supply a new model for us to explore the function of mitochondrial ferritin.

## 4. Materials and Methods 

### 4.1. Animals 

Mice were kept in accordance with the National Institutes of Health Guide for the Care and Use of Laboratory Animals, and by approval of the Animal Care and Use Committee of the Hebei Science and Technical Bureau, and the Laboratory Animal Ethical and Welfare Committee (AEWC) of Hebei Normal University (8 March 2015, Number: 2015-003). Mice were housed in stainless steel cages at 21 ± 2 °C and provided free access to food and water. The rooms maintained a 12 h light and 12 h dark cycle [45]. Age-matched wild-type male mice and FtMt overexpressing male mice (5 months old) were used in this study. 

The primers that we used to identify the genotype of mice were as follows:

Transgene PCR primer forward 5′-CCCACTGCTTACTGGCTTATCGAA-3′, and reverse 5′-TACACGTAGGATGCGTAAAGCTC-3′. 

Internal control PCR primer forward 5′-CAACCACTTACAAGAGACCCGTA-3′, and reverse 5′-GAGCCCTTAGAAATAACGTTCACC-3′. The internal control PCR targets the endogenous mouse Rgs7 (G protein signaling 7) locus, which exerts the role as internal primer to exclude the influence of the mouse itself.

The mice were anesthetized with pentobarbital sodium (40 mg/kg) and then were perfused with 0.9% saline. The body weights of the mice were measured before they were killed. The peripheral tissues (heart, liver, spleen, kidney, testis, thymus) were separated and washed in normal saline solution, dried with sterilized filter paper, and then weighed to calculate the organ coefficient. The tissues were later used to measure iron concentration. The brains of the mice were dissected into cerebral cortex, hippocampus, and striatum, and used for western blot and iron concentration analyses. 

### 4.2. RNA Isolation and Real-Time PCR

Liver hepcidin expression was measured by real-time PCR as previously described [44]. Total RNA was extracted from the liver with a TRIzol reagent (Ambion, Carlsbad, CA, USA) and 2 μg of the total RNA was reverse transcribed in a 20 μL reaction using TransScript One-Step gDNA Removal and cDNA Synthesis SuperMix (Transgen Biotech, Beijing, China) according to the manufacturer’s instructions. Control reactions without reverse transcription were performed to ensure that PCR products did not represent an amplification of genomic DNA. The solution was diluted to 100 μL, and 4 μL cDNA was then used as the template for real-time PCR with SYBRGreen (KangWei, Beijing, China). PCR amplification was performed with the BIO-RAD CFX Connect Real-Time System (Hercules, CA, USA) with the following parameters: 95 °C for 10 min, followed by 95 °C for 15 s, 60 °C for 1 min; 40 cycles. Each sample was repeated three times and the data were averaged. The primer sequences used for the PCR reaction were as follows:

Mouse hepcidin forward: 5′-AGACATTGCGATACCAATGCA-3′, and reverse: 5′-GCAACAGATACCACACTGGGAA-3′, *β*-actin forward: 5′-AGGCCCAGAGCAAGAGAGGTA-3′, and reverse: 5′-TCTCCATGTCGTCCCAGTTG-3′.

### 4.3. Hematological Analyses

Blood samples (50 μL) were collected from each mouse after anaesthetizing with 8% pentobarbital sodium. An anticoagulant was used to prevent blood clotting. Quantitative determinations of blood indices were performed using a BM830 automatic blood cell analyzer (Bao Ling Man Technology Company, Beijing, China). 

### 4.4. Blood Pressure and Heart Rate Measurement

Blood pressure and heart rate were measured in conscious animals using the tail-cuff method (CODA-2, Kent Scientific, Torrington, CT, USA). Briefly, mice were acclimatized for 6 consecutive days by measuring blood pressure and heart rate before the final results were recorded and in each session 15 consecutive readings were recorded. On the data collection day, the average of readings were used for sytolic pressure, diastolic pressure, and also heart rate.

### 4.5. Measurement of Serum and Tissue Iron 

The total iron concentration of serum and tissues (heart, liver, spleen, kidney, testis) was measured using Serum Iron Assay Kit and Tissue Iron Assay Kit from the Nanjing Jiancheng Bioengineering Institute (Nanjing, China) according to the manufacturer’s instructions. The serum was obtained by retro orbital bleeding, and the supernatant was collected after centrifugation at 2000× *g* for 20 min at 4 °C. 

### 4.6. Measurement of Brain Iron 

The total iron content of the brain regions (cerebellum, cortex, hippocampus, and striatum) was measured by inductively coupled plasma mass spectrometry (ICP-MS) as previously described [46]. The tissues used for the experiment were incubated for at least 12 h at 106 °C to dry tissues [47]. Before the experiment, the Teflon digestion tubes were washed with tap water, then double distilled water, and finally deionized water. The tubes were then soaked in 15% nitric acid for 24 h, washed with deionized water and then rinsed with ultrapure water. Approximately 6 mg sample was added to 1.5 mL ultrapure nitric acid (69.9–70.0%; J.T. Baker, Phillipsburg, NJ, USA), and then digested using a microwave digestion system for 2 h at 100°C and then 4 h at 200 °C. The completely digested samples were diluted to 2.5 mL with deionized water. Standard curves ranging from 0 to 100 ppb were prepared by diluting an iron standard (1 mg iron/mL) with blanks prepared from homogenization reagents in 0.2% nitric acid [48].

### 4.7. Western Blot Analysis

Protein expression was assessed by western blot as previously described [49]. The tissues were homogenized in a RIPA buffer containing 1% NP40 and protease inhibitor cocktail tablets (Roche Diagnostics GmbH, Roche Applied Science, 68298 Mannheim, Germany). The supernatant was collected after centrifugation at 12,000× *g* for 20 min at 4 °C, and the protein content was measured using a Bicinchoninic acid (BCA) Protein Quantification Kit (Yeasen Biotechnology, Shanghai, China). The extract, containing 40 μg of protein, was diluted in a 2× sample buffer, and heated for 5 min to denature the protein. The samples were resolved by SDS-PAGE, and then transferred to nitrocellulose membranes. The blots were blocked in 5% nonfat milk containing 20 mM Tris-buffered saline solution (pH 7.6, 137 mM NaCl, and 0.05% Tween-20; TBS-T) for 1.5 h at room temperature, followed by incubation with FtMt (Abcam Inc., San Francisco, CA, USA), TfR1 (ThermoFisher, Waltham, MA, USA), L-Ferritin, H-Ferritin (Abcam Inc., USA), DMT1(+IRE), DMT1(−IRE) (Alpha Diagnostic Intl Inc., San Antonio, TA, USA) or β-actin (Alpha Diagnostic Intl Inc.) primary antibody overnight at 4 °C. After four washes with TBS-T, the membrane was incubated with an anti-rabbit (Ruiying Bio, Suzhou, China) or anti-mouse (Ruiying Bio, Suzhou, China) horseradish peroxidase-conjugated secondary antibody for 90 min at room temperature. The specific proteins were detected by enhanced chemiluminescence (ECL), and analyzed with ImageQuant (Fujifilm LAS4000, Tokyo, Japan). The relative band intensities of the proteins are presented in comparison to that of β-actin [46]. 

### 4.8. Locomotor Activity Test

The locomotor activity was tested by a zz-6 mouse autonomic activity tester (Chengdu Technology & Marker CO. LTD, Sichuan, China). Locomotor activity counts and the stand-up times of mice were tested by infrared array beam. The mice were placed in the testing chamber (330 mm × 100 mm × 110 mm) to adapt to the environment before the experiment. Five mice from each group were tested for ten minutes per day, for four days. A quiet environment was required during the experiment.

### 4.9. Statistical Analysis 

All data are presented as the mean ± SEM. The differences between means was determined by one-way ANOVA, followed by post hoc Tukey tests corrected for multiple comparisons. Differences were considered significant if *p* < 0.05. All tests were performed using SPSS 21.0 (IBM SPSS21.0, Armonk, New York, NY, USA).

## 5. Conclusions

Our current study shows that FtMt overexpressing mice do not have an overt phenotype and that iron metabolism is not significantly influenced.

## Abbreviations

FtMt	Mitochondrial ferritin
PD	Parkinson’s disease
AD	Alzheimer disease
ROS	Reactive oxygen species
IRE	Iron responsive element
IRP	Iron regulation protein
SEM	Standard error of the mean
RBC	Red blood cell count
HGB	Hemoglobin
HCT	Hematocrit
MCH	Mean corpuscular hemoglobin
MCHC	Mean corpuscular hemoglobin concentration
MCV	Mean corpuscular volume
RDW-CV	Coefficient variation of red blood cell volume distribution width
ICP-MS	Inductively coupled plasma mass spectrometry
FPN	Ferroportin
TfR1	Transferrin receptor protein 1
DMT1	Divalent metal transporter 1
